# Drought Stress Response in Guar (*Cyamopsis tetragonoloba* (L.) Taub): Physiological and Molecular Genetic Aspects

**DOI:** 10.3390/plants12233955

**Published:** 2023-11-24

**Authors:** Margarita A. Vishnyakova, Nadezhda Frolova, Andrej Frolov

**Affiliations:** 1 N. I. Vavilov All-Russian Institute of Plant Genetic Resources (VIR), Saint-Petersburg 190000, Russia; 2 Laboratory of Analytical Biochemistry and Biotechnology, Timiryazev Institute of Plant Physiology, Russian Academy of Sciences, Moscow 127276, Russia; frolovanadja@yandex.ru

**Keywords:** guar, *Cyamopsis tetragonoloba* (L.) Taub, drought stress, genotypic variability, metabolome and transcriptome changes, physiological and molecular markers, productivity

## Abstract

Drought has become one of the main factors of crop yield losses worldwide. This negatively affects the plant industry, decreasing crop yields, and it may result in resource deficits in different sectors of the world economy and its national branches. Guar (*Cyamopsis tetragonoloba* (L.) Taub) represents one of the strategic crops, as its seeds are the source of guar gum, which is critically important in the modern oil industry. Although guar is generally known to be a drought-tolerant plant, it is known that soil dehydration negatively affects plant fitness and crop productivity. As guar genotypes are characterized by high variability in the manifestation of drought tolerance, screening genetic resources for this feature seems to be a promising strategy for accessing drought-resistant varieties. The discovery of drought-tolerant genotypes is mandatory to secure sustainable guar production. In this context, the identification of reliable chemical and molecular markers of drought tolerance (i.e., drought-responsive and/or drought-protective metabolites, proteins and transcripts) will provide the solid basis for marker-driven breeding of new tolerant varieties. Therefore, here we provide a comprehensive overview of the available literature data on guar drought stress response, its physiological and molecular genetic aspects, and considerations on the approaches to improve the quality of this crop.

## 1. Introduction

Guar (*Cyamopsis tetragonoloba* (L.) Taub.) is an annual grain legume crop. Due to its strategic significance, it is recognized as one of the most sought-after and promising crop plants in the world. To date, the main rationale for the cultivation of guar is the high abundance of galactomannans (also known as guar gum or guarana) accumulated in the endosperm of its mature seeds [1]. As a plant polymer built from individual monosaccharides (galactose), guar gum behaves as a hydrocolloid, i.e., when dissolved in water and other liquids, it is capable of forming gelatinous mixtures resistant to freezing. This property has a high demand in the food, medical, textile, paper, cosmetics and explosives industries. However, the most important aspects of its application are the oil production and mining industries [2]. As a drilling fluid additive, guar gum has unrivaled properties [3]. When used during oil drilling, guar gum prevents the loss of water from viscous drilling fluid and effectively suspends bentonite clay. Guar gum is also cheaper than most other drilling fluid thickeners. The high worldwide demand for guar in recent decades has been the impetus for closer study and breeding of this crop in the countries of its predominant cultivation: India, the USA, Pakistan, Australia and several southern European countries. The Russian Federation, along with Germany, the Netherlands, Italy, France, Spain and the UK, is the largest importer of guar gum in Europe [4].

Dependence on the guar import motivated European and Russian scientists and agrarians to initiate the work on the introduction of this tropical crop in the industrial cultivation in their countries. Over the last decade, significant successes in guar cultivation have been achieved in several regions of the Southern Federal District and the Lower Volga region in Russia [5]. Moreover, the State Register of Breeding Achievements was extended with ten cultivars of guar, nine of which are of domestic breeding [6]. This fact is expected to impact the development of domestic gum production. Guar can also be used for animal feed and as green manure [7]. Similarly to other legumes, it forms symbiotic interactions with nitrogen-fixing rhizobia and arbuscular mycorrhiza fungi [8,9]. Therefore, the introduction of this legume crop in agricultural production in Russia will contribute to the development of green agriculture and the technological independence of the national oil production industry. Obviously, a proper soil type and climate zone need to be selected for the successful cultivation of guar in Russia [5]. For this, the mechanisms behind the adaptation of guar plants to soil and environmental conditions need to be comprehensively addressed, in addition to the diversity of their gene pool with respect to limiting environmental factors.

In particular, it is important in the context of drought, which is typical in the southern regions of the Russian Federation. Drought is known as the main factor affecting crop productivity worldwide [10] and requires, therefore, the special attention of agricultural biologists. In respect of this factor, guar is generally considered a moderate drought- and heat-tolerant crop [11,12]. However, the intensive studies of the recent decade [13,14,15] clearly indicated that individual guar genotypes are featured with a pronounced diversity in responsivity to different irrigation modes: cultivation without irrigation, with variable water supply, as by quantity, during different stages of plant development.

Therefore, guar breeding might potentially rely on a differentiated (multitarget) approach. In terms of this approach, drought-tolerant cultivars can be cultured under water deficit conditions, whereas drought-sensitive ones can be adjusted to a specific water supply regimen. Obviously, under the climate conditions of the Russian Federation, the availability of elevated temperatures (25 °C and more) appears to be the principal limiting factor for guar field culturing. Indeed, for this crop, the sum of efficient air temperatures above 10 °C must be at least 3400–3500 °C. In terms of rainfall, 350–500 mm per growing season was reported to be sufficient [16]. Expectedly, only a few Russian regions meet these criteria. Thus, the plain parts of the Stavropol and Krasnodar regions have a natural water supply, which can be judged as sufficient for guar culturing, whereas supplementary irrigation is recommended in Crimea and in the Rostov region [16,17]. Therefore, searching and discovering drought-tolerant genotypes among the available guar gene pool is strongly mandatory for the successful cultivation of the crop in arid regions of southern Russia [5]. Taking into account the role of the oil industry in the national economy of Russia, this aspect remains highly relevant. However, to accomplish this task successfully, the physiological and biochemical mechanisms behind the drought tolerance in guar need to be well understood, and the underlying molecular genetic aspects of this trait need to be disclosed. Despite the high importance of water regimen in culturing legumes, for guar, this aspect still remains mostly unstudied, which might be explained by the limited genome and transcript information available for this species [18]. Therefore, in this review, we summarize the available literature data on the physiological mechanisms behind the drought tolerance of guar, as well as the use of genomic and transcriptomics data for understanding the regulatory and metabolic pathways underlying drought tolerance.

## 2. Drought Stress Response in Guar (*Cyamopsis tetragonoloba* (L.) Taub.)

### 2.1. Biology of Guar

Guar is an unpretentious plant and grows on both sandy and well-drained clay soils. Guar is self-pollinating with a negligible level of cross-pollination [19]. The plants considerably vary in height (from 50 cm to 1.5 m). The stem is sturdy, becoming woody by the plant maturation. The main root is thick and tapering in its distal parts, deeply penetrating into the soil. Due to this, guar can perfectly sustain short-term drought. In the center of its origin—India [20]—guar is cultivated predominantly in the non-irrigated arid areas of the north-west of the country in the Thar Desert, which also covers the south-east of Pakistan. This area receives only 90–200 mm of rainfall per year, almost all during the summer monsoon (from July to September). However, it should be noted that varieties, destined for food consumption, are only grown on irrigation [21].

The guar pods (beans) are arranged in heaps, forming groups (clusters). Due to this fact, the original English name of this crop is cluster beans. The beans are straight, slightly curved, 4 to 14 cm long, with 5 to 12 seeds in a pod. The embryo occupies approximately 40–45% of the seed volume, the seed coat corresponds to 14–16%, and the endosperm (which contains galactomannan as the main non-protein reserve polymer) accounts for 38–45% of the seed [22]. Galactomannan, in turn, accounts for 75–80% of the endosperm mass [23].

### 2.2. Guar as a Drought-Tolerant Plant

#### 2.2.1. Genotypic Variability of Guar in Respect of Drought Tolerance

Although guar is universally considered a drought-tolerant plant, the information about the effects of water deficit on its growth and productivity is limited. By varying the dates of guar sowing in Sicily for two years (7 May and 26 June 2003, 18 May and 30 June 2004), the average value of the rainfall falling there during the guar growing season was established: 2160 m^3^/ha for late sowing and 3150 m^3^/ha for early sowing, due to rainfall typical of September and October [24]. Similar data for Sicily—3000 m^3^/ha during the guar growing season—have been shown by other researchers [25].

When the strategy of limited water supply was applied in Sicily [15], different irrigation modes were addressed with five American guar cultivars: Kinman, Lewis, Matador, Monument and Santa Cruz. Specifically, the following irrigation modes were used: full (FI, 100% soil moisture capacity), medium (MI, 50%) and low irrigation (LI, 25%). The result of the limited water supply was a visible decrease in the number of beans and seed mass per plant, plant height, leaf area index. Seed yields ranged from 1.24 (LI) to 3.28 tons/ha-1 (FI) in 2011 and from 0.98 (LI) to 2.88 tons/ha-1 (FI) in 2012. Compared to FI, the two-year average reduction in seed yield for the LI and MI variants was 49% and 26%, respectively. The varieties Lewis and Santa Cruz showed significantly higher seed biomass yields under the conditions of both full and limited irrigation. This fact might indicate the tolerance of guar plants to limited water supply. The highest values of water use efficiency were observed under the FI watering mode (1.44 kg/m^3^ with gains in water use efficiency of 34 and 95% compared to MI and LI, respectively).

Thus, it was reliably proven that guar has a good potential for high crop yields at sufficient water supply, although it is still able to maintain acceptable yields under the conditions of limited water supply. Indeed, a 50% water deficit reduced crop yields by not more than 26%, indicating high efficiency of water use by guar plants under conditions of low water availability [15]. This observation was confirmed by the works of Stafford and McMichael [26], who showed that the parameter of yield most affected by water stress was the number of pods/plants, which decreased significantly. Seed weight, seeds per pod and racemes per plant each had a progressively smaller impact on the seed yields. Thereby, the number of pods per plant was most negatively affected by drought, whereas the number of seeds per pod appeared to be the least responsive trait [15,24]. The experiments of Alshameri and co-workers [27] accomplished in Pakistan showed that the drought-induced decrease in the number of pods formed was accompanied by a drop in the number of leaves, leaf area, leaf dry weight, total shoot biomass and plant height.

Recently, several studies were accomplished to address the stages of guar ontogenesis, which are the most sensitive to water stress. It was shown that guar plants are most susceptible to deleterious effects of drought during the reproductive stage, namely during flower formation and flowering [15,28,29,30]. The genotypic variability in the response of guar plants to drought stress noted in Avola et al. [15] has been noted by other researchers. In the gene pool of the crop, as in most agricultural plants, there are genotypes with varying degrees of drought tolerance.

This fact can be illustrated by the work of Ali and co-workers [13], who compared the water stress responses of 36 guar cultivars grown in the arid climate of Pakistan. The authors subjected 20-day-old plants to water stress by interruption of watering for 20 or 40 days. Thereby, fresh and dry plant biomass, height, CO_2_ assimilation rate, transpiration rate and air CO_2_ concentrations were addressed. The pronounced drop in all these parameters was observed for all guar cultivars even upon medium-term (20-day-long) watering interruption. However, the degree of these changes varied essentially, i.e., the most drought-tolerant plants could be identified [13].

Such genotypic variability was confirmed in the study of Khandaza et al., who introduced 24 Pakistani guar cultivars as a fodder crop in Saudi Arabia [31]. This study revealed variability in responses to heat, drought and soil salinization. Thereby, shoot weight and freshness at the irrigation level corresponding to 100% (control), 80%, 60% and 40% of the field water supply capacity served as indicator traits. Based on the observed variability in drought stress responses, cultivars contrasting the trait “drought tolerance” could be identified [31].

Inspired by these data, we accomplished a comprehensive literature mining to reveal the lines/cultivars reported earlier as drought-tolerant (Table 1). For this, we analyzed original literature on individual accessions present in different gene banks worldwide.

To study the mechanisms of stress resistance, genotypes contrasting in the manifestation of the trait are used. Therefore, in Table 2, we present some drought-susceptible accessions from the same gene banks. The information about these lines/cultivars is gleaned from the works of the authors cited above.

Recently, a comprehensive ecological and geographic screening of guar germplasm was accomplished in the Russian Federation [36]. The study included the cultivation of 13 cultivars in locations where climatic conditions corresponded to the temperature requirements of the crop on irrigated lands and on lands where irrigation was not applied. The experiments revealed a multi-fold increase in seed productivity and gum yields under optimal water supply (i.e., under sufficient irrigation).

However, no experiments aiming at the identification of drought-tolerant cultivars produced by Russian breeders have been conducted so far. Thus, prospective studies aiming identification the optimal guar irrigation modes to access maximal possible productivity are strongly mandatory. These studies would also deliver important information on drought-tolerant genotypes. This will allow the formation of a pool of genetic resources for breeding new promising drought-tolerant guar varieties.

#### 2.2.2. Physiology of Drought Stress Response of Guar with Respect to Drought Tolerance

Taking into account the dynamics of ontogenetic changes and the whole plethora of biotic and abiotic plant interactions with the environment, the general picture of plant response to drought stress appears to be quite complex. Typically, these responses rely on several major strategies and involve multiple levels of life organization—from the cellular and sub-cellular to the organism one [36].

The first plant strategy, usually referred to as “drought escape”, develops in response to short-term water deficit. According to this strategy, plants complete their life or growth cycle before the onset of drought-related deleterious effects and cellular damage [37,38]. Longer exposure to dehydration triggers another strategy of plant response to drought, “drought avoidance” [39]. This strategy relies on maintaining the water potential (Ψ_w_) of tissues at the physiologically acceptable level by increasing water uptake or limiting water loss [40]. In the early stages of the drought response, this is mainly achieved by stomata closure (and, hence, a dramatic decrease in transpiration), which is triggered by abscisic acid (ABA). Due to this, the plant water losses via transpiration, as well as during gas exchange (related to photosynthesis and respiration), are reduced [39,41,42]. In addition to ABA, reactive oxygen species (ROS) and calcium ions (i.e., redox and Ca^2+^-signalling) play a key role in the regulation of stomatal closure. A key role in the regulation of stomatal closure is played by the synthesis of CLE25 (CLAVATA3/EMBRYO-SURROUNDING REGION RELATED 25) peptide in response to water deficit, which is transported into guard cells and promotes ABA synthesis. ABA-activated SnRK2 protein kinase promotes apoplastic production of ROS outside of guard cells and its transport into guard cells. Apoplastic H_2_O_2_ is directly sensed by the receptor kinase HPCA1 (HYDROGEN PEROXIDE-INDUCED CA^2+^ INCREASES1), which induces activation of calcium channels in the cytomembrane of guard cells and causes an increase in Ca^2+^ in the cytoplasm of guard cells, leading to closure of the stomata [43,44,45]. In roots, ABA increases the permeability of root hairs to water and enhances root elongation growth [38]. This allows more efficient use of restricted water resources and enhances the assimilation of water found in deeper soil layers [38]. Redirection of assimilated transport also contributes to accelerated root growth [46].

When drought persists for a long time, and the adaptive capacity of the “drought escape” and “drought avoidance” strategies is insufficient to maintain plant growth and productivity, drought tolerance mechanisms can be activated [47]. At this stage, a profound rearrangement of plant metabolism occurs, and mechanisms such as accumulation of osmoprotective proteins—so-called metabolic adjustment; cell wall reinforcement; ROS detoxification; and an increase in the contents of osmolytes, primary metabolites, predominantly amino acids and sugars—may be involved [36,48,49,50]. Along with the complex regulatory mechanisms of antioxidant defense, carbohydrate and nitrogen metabolism, including the control of gene expression and the activity of key enzymes [33], significant changes in the composition of osmoprotective metabolites and proteins associated with the drought onset are crucial for plant survival [39,51]. Therefore, targeting the mechanisms of drought tolerance by the methods of plant physiology, molecular biology and post-genomic techniques might provide access to new strategies of plant cultivation and breeding to obtain new drought-tolerant cultivars.

### 2.3. Mechanisms behind the Guar Drought Tolerance

#### 2.3.1. Effects of Drought on Photosynthesis and Carbohydrate Metabolism

Photosynthesis is a vulnerable physiological process, usually dramatically affected by drought. These effects are typically manifested with the degradation and denaturation of chloroplast membrane systems [52]. Drought-induced suppression of photosynthetic activity is typically associated either with a restriction of the stomatal uptake of carbon dioxide and its transport to mesophyll cells, with enhancement of oxidative stress, or with inhibition of photosynthetic enzymes [53,54]. Moreover, drought results in disruption of respiration and associated oxidative phosphorylation that results in inhibition of ATP synthesis [55,56]. To understand these metabolic changes, adequate assessment of the drought-induced alterations in the structure and function of photosynthetic apparatus is required. The most adequate (although indirect) approach to address these changes in drought-sensitive guar plants is the assessment of the leaf water status and gas exchange efficiency. In particular, besides the evaluation of their gas exchange parameters, the determination of spectral characteristics (e.g., photochemical reflectance index (PRI) and normalized difference vegetation index (NDVI) are advantageous [57]. On the other hand, the optical properties of leaves need to be considered. Indeed, the ability for dynamic modification of their optical properties (i.e., reflection, absorption and transmission of light by the leaf lamina) represents one of the most important leaf adaptations [58]. Dynamic characterization of the drought-induced shifts in these properties in parallel to the analysis of gas exchange parameters and leaf pigment contents showed that the decrease in leaf photosynthetic activity was mostly associated with such markers as leaf relative water content (LRWC), stomatal conductance and drop in chlorophyll contents [59,60]. In contrast, a drought-induced increase in the contents of carotenoids and anthocyanins, which was most distinctly observed in the drought-tolerant guar line RGC-1002, impacted the improvement of membrane integrity and inhibition of formation. These biochemical shifts ensured the protection of the photosynthetic apparatus from light stress by absorbance of high-energy light. Moreover, the line RGC-1002 demonstrated a stress-induced increase in the NDVI index, which might indicate an increase in the maximal absorption of red light by chlorophyll molecules [57]. Upreti et al. found that by characterizing various chlorophyll fluorescence parameters, variety RGC-1002 has a better ability to dissipate excess light energy harmlessly compared to the drought-sensitive cultivar RGC-936 and thereby attenuate photoinhibition of both photosystems and protect the photosynthetic apparatus under drought conditions. The decrease in photosynthesis under drought conditions was smaller in the RGC-1002 cultivar compared to RGC-936, and the discrimination of stable carbon isotopes decreased more strongly in RGC-1002 [35].

To ensure the availability of energy resources (which are actually restricted under drought conditions) for essential biochemical reactions of cell primary metabolism involved in stress adaptation, plants employ the storage-mobilization strategy [61,62]. In this context, the processes of photosynthesis and different branches of carbohydrate metabolism need to be tightly coordinated. A study of carbohydrate metabolism and photosynthesis in drought-tolerant guar cultivars RGC-986 and HG-563 compared to the sensitive ones, RGC-471 and Varsha, revealed a drought-induced decrease in photosynthetic rates in all the cultivars studied, resulting in an apparent decrease in the contents of nicotinamide adenine dinucleotide phosphate (NADPH), which is required for CO_2_ assimilation in the Calvin cycle [33]. Drought tolerant cultivars RGC-986 and HG-563 were found to exhibit more efficient coordination at the level of the photosystem II (PS II) and Calvin cycle gene regulation compared to drought tolerant cultivars RGC-471 and Varsha; higher efficiency of ROS detoxification; and enhanced biosynthesis of glucose, fructose and fructones associated with drought-protective guar metabolic rearrangement [33].

It is important to note that glucose is not the only drought-inducible osmoprotective compound reported in guar so far. As was shown recently, guar plants synthesize fructans, which act as osmotically active compounds also involved in the protection of cellular membranes from damage and impact on maintaining the turgor pressure [63]. The main factor affecting the efficiency of fructan biosynthesis in guar leaf cells is the availability of sucrose, the substrate of the enzyme sucrose: sucrose fructosyltransferase (SST), which irreversibly catalyzes the transfer of fructosyl between two sucrose molecules to form 1-kestose and glucose [64]. The ketose intermediate can serve as an acceptor for fructosyl residues, which can be transferred from another fructan chain, serving as a donor in the chain elongation reaction catalyzed by fructan: fructan fructosyltransferase (FFT) [64,65]. Stress-induced enhancement of photosynthetic reactions results in enhancement of sucrose biosynthesis [66,67]. Further, the enhanced rates of 1-kestose formation and subsequent chain elongation in transfructosylation reactions support the intensive synthesis of fructose-containing oligosaccharides, leading to their accumulation in the vacuole [68]. However, the molecular mechanisms underlying carbohydrate transport in response to drought stress remain unclear. In particular, it was found that the active response to drought is the activation of the trehalose-6-phosphate/SNF1-linked protein kinase (SnRK1) signaling pathway by suppressing class I Trehalose Phosphate Synthase (*TPS*) and the expression of class II *TPS* genes. The expression of *SnRK1a* and *β-subunits*, as well as *Sucrose Synthase 6*, contributed to the accumulation of soluble sugars in the leaves, the accumulation of which in vacuoles supports osmoregulation in the leaves. The increased expression of sucrose synthesis genes and the reduced expression of sucrose degradation genes in the roots did not coincide with sucrose levels, which implies local sucrose production for energy [69].

The cellular fructose and sucrose pools are closely related metabolically, as they are in a continuous equilibrium state. This equilibrium is manifested by the intensive exchange of fructosyl residues, which accompanies both the accumulation and depletion of fructan storages [70]. Moreover, the pools of reserve fructans and starch are also dynamically interconnected via sucrose. Indeed, this metabolite serves as a substrate in the biosynthesis of both polymers [71]. Thus, concerted regulation of carbohydrate metabolism and photosynthesis in guar impacts the efficient survival strategy under abiotic stress [33].

Recently, Ansari and co-workers accomplished a comparative transcriptome analysis of guar cultivars characterized by different drought tolerance. This study revealed an increased level of transcripts related to starch and sucrose metabolism in the drought-tolerant guar genotype RGC-1002 [34]. This fact can be considered as further support for the drought-induced redirection of carbon flux to carbohydrate metabolism in guar leaves. Another drought-tolerant guar variety, BWP-5595, also demonstrated increased expression levels of three genes of the SWEET (Sugars Will Eventually Be Exported Transporter) family of sugar transporters [27,72]. This fact is also in line with the proposed concept.

#### 2.3.2. Effects of Drought on the Nitrogen Metabolism

Along with carbon metabolism, nitrogen metabolism is an important factor in plant growth, development and reproduction. Nitrogen assimilation is an energy-consuming process (especially when nitrate acts as the main nitrogen source) and is associated with high consumption of ATP [73]. Moreover, it is necessary to take into account the significant role of nitrogen consumption in photosynthesis [74]. Thus, in comparison to carbon assimilation, nitrogen assimilation appears to be a more critical factor for plant survival [75,76]. This becomes even more obvious under drought conditions when multiple enzymes of the nitrogen metabolism are inhibited, and carbohydrate accumulation in plant tissues is observed [77,78].

To meet the plant demand for both amino acid and carbohydrate biosynthesis [79], the assimilation of carbon and nitrogen in irradiated leaves needs to occur simultaneously and in a well-coordinated way, i.e., the relevant metabolic pathways need to be tightly regulated. Thereby, the tissue levels of reduced nicotinamide adenine dinucleotide (NADH) are critical for successful nitrate and ammonia assimilation [80]. Thus, the carbohydrate metabolism (including glycolysis and tricarbon acid cycle, TCA) yields appropriate amounts of ATP and provides a pool of reduced NAD(P)H nucleotides necessary for the reactions of nitrogen assimilation. It is important to note that besides the contribution to the ATP/NADH pool, TCA serves as the precursor of some amino acids (e.g., glutamate), which rely on the carbon skeleton of 2-oxoglutarate, a metabolite of the tricarboxylic acid (TCA) cycle [78].

Generally, regulation of the carbon-to-nitrogen ratio in plant tissues typically relies on the ammonium and nitrate ions, as well as several key nitrogen-containing metabolites such as glutamate, glutamine and aspartate [73]. These metabolic regulators interfere with cytokinin signaling, which, in turn, is involved in the regulation of plant growth responses to alterations in nitrogen supply availability [81]. Nitrate enhances the expression of the key enzyme of cytokinin biosynthesis—isopentenyltransferase IPT3. Cytokinin is perceived by receptors, and the signal is transmitted by phosphorylation of the two-component His-Asp system and subsequent cytokinin-mediated signaling associated with development control, protein synthesis and macronutrient assimilation [81]. In addition, experiments in conditions of water scarcity on transgenic IPT plants under the control of P(SARK), a promoter induced by maturation and stress, *Oryza sativa* japonica “Kitaake” demonstrated that stress-induced cytokinin synthesis contributed to increased uptake through cytokinin-dependent coordinated regulation of carbon and nitrogen metabolism, which contributes to increased tolerance in transgenic plants exposed to water deficit [82].

The short-term effects of drought on carbohydrate and nitrogen metabolism were addressed with the four contrasting guar cultivars described above, i.e., two drought-tolerant accessions (HG-563 and RGC-986) and two sensitive ones (RGC-471 and Varsha). The guar plants were subjected to 15 days of drought by watering interruption, after which such parameters as respiration, gas exchange efficiency, water potential, changes in activities of the enzymes involved in carbon and nitrogen metabolism along with expression of genes encoding the principal actors of oxidative phosphorylation, TCA cycle and nitrogen metabolism, were evaluated by an array of physiological, biochemical and molecular approaches [78]. Thus, nitrogen and carbon contents, assessed by the isotope-ratio mass spectrometry (IRMS)-based elemental analysis in the leaves, stems and roots of the four differentially drought-tolerant guar accessions, demonstrated a strong decrease and increase, respectively, in response to experimental drought in comparison to corresponding controls [78]. Thus, the nitrogen concentration decreased significantly (*p* < 0.05) at all stages of drought in all studied varieties of guar plants, while the carbon concentration significantly increased in all guar varieties exposed to water stress than in control plants. Roots decreasing in nitrogen concentration were observed, with 72% in Varsha variety, 68% in RGC-471, 45% in RGC-986 and 22% in HG-563 compared to control plants. The trend of decreasing nitrogen concentration continued in the stems and leaves of the analyzed varieties. In the roots and stems of the four varieties, HG-563 showed a higher concentration of carbon, followed by RGC-986, RGC-471 and Varsha, while in the leaves, at the stage of severe drought, the carbon concentration increased by 36% in HG-563, 31% in RGC-986, 21% in RGC-471 and 16% in Varsha varieties, respectively [78].

To date, analysis of the stress-induced patterns of differential gene expression represents one of the major approaches to addressing the mechanisms behind drought tolerance in plants. Recently, such patterns were reported for guar. Thus, the transcript levels of the genes related to nitrogen metabolism in *C. tetragonoloba* (nitrate reductase, nitrite reductase, glutamine synthetase and glutamate synthetase) demonstrated significant changes in response to drought. Specifically, the decrease in expression levels was more pronounced in the drought-sensitive guar varieties RGC-471 and Varsha, which hindered the growth and development of those plants [83].

#### 2.3.3. The Role of Tricarboxylic Acid (TCA) Cycle Metabolites in the Drought Tolerance of Guar

The impressing numbers and diversity of the individual metabolites and even metabolic pathways involved in the plant response to drought might indicate the high capacity of plants to adapt to environmental changes [84,85]. Thus, the pathways of the primary and secondary metabolism, such as glycolysis, TCA cycle, urea cycle, amino acid metabolism, glutamate-mediated proline biosynthesis, synthesis of phytohormones and unsaturated fatty acids, as well as the biosynthetic pathways of phenolic compounds, are critical for the plant response to drought. Among these, the TCA cycle represents a crucial aerobic pathway involved in the final steps of carbohydrate oxidation and plays a key role in establishing drought tolerance in plants [85]. To obtain a complete picture of the accompanying physiological changes, the expression levels of the genes encoding the key enzymes of oxidative phosphorylation and tricarboxylic acid cycle (which are assumed to be indicative of the effect of drought on the respiratory metabolism) were analyzed in guar plants [78,83].

In the most efficient way, this aspect can be considered in the context of nitrogen metabolism. Thus, recently, a comparative study of the guar carbon (oxidative phosphorylation and tricarboxylic acid cycle) and nitrogen metabolism revealed increased expression levels of the genes involved in respiration-related metabolic pathways and decreased expression levels of the genes encoding enzymes of nitrogen metabolism pathways involved at all stages of the plant response to drought [80]. The expression levels of pyruvate dehydrogenase (*PDH*), which is involved in the oxidative decarboxylation of pyruvate, gradually increased in guar tissues as drought response developed in the stressed plants in comparison to the controls. The transcript levels of the gene encoding phosphoenolpyruvate carboxylase (*PEPC*), which catalyzes the interaction between bicarbonate and phosphoenolpyruvate to yield oxaloacetate and inorganic phosphate, were increased under drought conditions in all four guar varieties. Thereby, the most pronounced stress-induced increase in the levels of the *PEPC* gene expression was observed in the drought-tolerant cultivar HG-563, whereas the drought-sensitive cultivar Varsha showed the least expressional response. The maximal expression level of dihydrolipoamide dehydrogenase (*DLD*), a mitochondrial enzyme that plays a vital role in energy metabolism in plants, was also observed in drought-tolerant guar variety HG-563 [78,83]. The upregulation of DLD was maximum in HG-563 by about 4.2 times in comparison to control plants at severe stages of drought [78]. DLD converts dihydrolipoic acid and NAD+ into lipoic acid and NADH. Lipoic acid is an antioxidant that also stimulates photosystem II activity and the gene expressions of carbon fixation and chlorophyll metabolism enzymes [86].

Also, in the study by Pandey and co-workers [78], it was found that the leaf tissue levels of aspartate aminotransferase (*AspAT*) and isocitrate dehydrogenase (*ICDH*) activities were decreased under drought stress. On the other hand, a pronounced stress-induced increase in the aminase and glutamate deaminase (*NADH-GDH/NAD-GDH*) activities in all the varieties analyzed. Other important genes involved in mitochondrial respiration—NADH dehydrogenase (*NADH DEHYDROGENASE*), cytochrome C oxidase (*CYT C OXIDASE*), cytochrome C reductase (*CYT C REDUCTASE*) and pyrophosphorylase *(PPP*), showed increased transcript levels in all four guar cultivars exposed to drought: HG-563, RGC-986, Varsha and RGC471. Thereby, the levels of all transcripts were higher in the drought-tolerant cultivars HG-563 and RGC-986 in comparison to the drought-sensitive ones RGC-471 and Varsha [78,83].

Generally, the enhancement of cellular respiration is thought to be utilized by the plant as a short-term drought adaptation mechanism to cope with the demand for increased energy consumption [87]. In agreement with this, under the drought conditions, all studied guar cultivars showed pronounced changes in the transcript levels of the key TCA components, which likely serve as the targets in the biochemical rearrangement behind the stress-induced metabolic adjustments. Specifically, a decrease in the 2-oxoglutarate dehydrogenase (*OGDH*) gene expression level, along with up-regulation of succinate dehydrogenase (*SDH*) and mitochondrial malate dehydrogenase (*MDH*) transcripts, were observed. It was also found that the expression levels of the fumarate dehydrogenase (*FDH*) gene were increased in the guar varieties HG-563 and RGC-986 and decreased in Varsha and RGC471 [78,83].

Thus, changes in the expression of the genes encoding TCA enzymes and alterations in the abundances and/or activities of the corresponding key enzymes indicate the effect of drought on respiratory metabolism. These findings support increased energy requirements of guar plants and increased rates of respiratory reactions under stress conditions.

#### 2.3.4. Effect of Drought on the Guar Antioxidant Defense

It is well known that dehydration of plant tissues triggers oxidative stress, i.e., the state when the capacities of the cellular antioxidant systems are overwhelmed by enhanced generation of reactive oxygen species (ROS) [88,89]. ROS are highly reactive and cause severe damage to membranes and disruption of their functions, as well as inhibition of multiple enzyme activities, enhancement of mutagenesis and cell cycle arrest, leading to the death of individual cells or even the whole organism [52,90]. Detoxification of ROS in plants is accomplished by the antioxidant defense system, which includes both enzymes (peroxidase, catalase, ascorbate peroxidase, ascorbate peroxidase, superoxide dismutase, glutathione reductase) and small molecules (ascorbic acid, glutathione, cysteine, proline) [91].

In agreement with this, the drought-induced oxidative stress was reported to cause an increase in the activities of superoxide dismutase (SOD), catalase (CAT) and ascorbate peroxidase (ASP) in the drought-tolerant guar cultivar RGC-1002. Interestingly, although the activities of glutathione reductase (GR), ascorbate peroxidase (APX) and dehydroascorbate reductase (DHAR) were increased in dehydrated leaves of these plants, the corresponding activities were only minimally affected when water stress was applied to drought-sensitive cultivar RGC-936. Moreover, the RGC-1002 plants were featured with higher levels of tissue phenolics and proline in comparison to the drought-sensitive guar variety RGC-936, although the levels of the oxidative stress marker malondialdehyde and the total phenolic contents (well-known indicators of the drought-related cell damage) were increased in the tissues of all cultivars addressed in the experiment [35].

Regarding the low molecular weight antioxidants, drought triggers a pronounced enhancement in the synthesis of small antioxidant molecules like ascorbic acid (ASC) and glutathione (GSH). Thereby, the ratios of ascorbic acid/dehydroascorbate (ASC/DHA) and reduced/oxidized glutathione (GSH/GSSG) are typically increased in response to dehydration. Thus, in another study, the maximal increases in these ratios (nine- and eight-fold, respectively) were found in the drought-tolerant guar cultivar RGC-1002. In the drought-sensitive cultivar RGC-1066, the ratio of both these antioxidants was significantly reduced [34].

#### 2.3.5. Effect of Drought on the Metabolism of Phytohormones in Guar

Plant response to abiotic stress (particularly to drought stress) relies on a complex array of physiological, biochemical and metabolic reactions in which phytohormones play an essential role [34]. The early responses are at least partly associated with the cellular damage and are represented by a pattern of relatively fast and efficient adaptations. These adaptations allow for preserving cell functionality under water stress conditions and are typically manifested with metabolic adjustment and alterations in cell structure [92].

For example, the contents of abscisic acid (ABA) are well known to increase under drought stress conditions [38]. This metabolic shift protects plants from tissue dehydration by triggering stomata closure [93]. This effect is underlined by an array of ABA-induced transcriptional regulatory responses. Thus, drought-tolerant guar cultivars HG-563 and RGC-986 showed increased (in comparison to the stress-sensitive ones RGC-471 and Varsha) expression of F-box and WRKY transcription factors, which regulate ABA-mediated responses to drought [94].

Besides ABA, several other hormones and corresponding associated signaling pathways affect the control of stomatal conductivity under water deficit conditions. While brassinosteroids, jasmonic and salicylic acids support ABA effects, auxins, cytokinins and ethylene tend to inhibit the ABA-mediated mechanism of stomatal closure [36]. Besides the ABA signaling, the WRKY transcription factor is involved in the regulatory network associated with the salicylate and jasmonate signaling induced in response to the osmotic stress [95].

### 2.4. Genomics and Post-Genomics Techniques in the Study of Drought Tolerance in Guar

To understand the events accompanying the onset of drought tolerance and to address the underlying molecular mechanisms, an integrated physiological and biochemical approach is required. Therefore, the biochemical component needs to rely on the methods of genomic and post-genomic research. However, to date, only a little information on the identification and functional characterization of the guar genes and proteins involved in growth, development and stress tolerance is available [96,97]. Although the degree of drought tolerance varies significantly among the guar genotypes, the metabolic features underlying these differences at the metabolome and proteome level remain poorly understood. To date, predominantly genomics and transcriptomics approaches were successfully employed in the study of guar genetic resources [34,72,98,99], whereas proteomics and metabolomics studies are still to be conducted.

#### 2.4.1. Guar Genome Resources for Breeding of New Prospective Drought-Tolerant Cultivars

From the point of genomics, guar remained principally unstudied until the beginning of the 21st century (late 2010s), when Tyagi and co-workers determined the size of the guar genome as 581 Mb by the flow cytometry analysis [100]. The chloroplast genome of guar, which was estimated to be 152,530 bp, was sequenced and compared with some previously reported chloroplast genomes of legumes [101]. This knowledge provided deeper insights into some evolutionary and molecular features of this plant. Recently, the first genome assembling study was accomplished with the guar cultivar Vavilovsky 130 [102]. The authors combined the information obtained from short and long reads by the Illumina and Oxford Nanopore platforms, respectively. This work yielded successful assembly of the reference genome with 50% coverage of the entire guar sequence. According to the obtained genome sequence data, guar appeared to be phylogenetically close to the genera *Vigna*, *Abrus*, *Glycine* and *Lupinus*. However, for efficient use of the world’s genetic resources in breeding programs, a complete and high-quality reference genome of guar is still necessary.

Despite the incompleteness of the currently available reference genome, several reliable molecular markers could be identified in guar. The most remarkable of them are RAPD (random amplified polymorphic DNA), AFLP (amplified fragment length polymorphism), SSR (simple sequence repeats), SNP (single-nucleotide polymorphism) [18] and SCAR (sequence characterized amplified region) [103]. All these markers are intensively used in breeding.

Thus, several agronomic traits, like growth patterns and the number of seeds per pod and beans per plant, were addressed as AFLP-related breeding markers [24], whereas SSRs were recently employed in the studies of guar genetic diversity [98]. Also, Grigorieva and co-workers proposed SNPs, which were potentially efficient as markers associated with the traits underlying high seed productivity (i.e., the number of matured beans on the plant) of guar plants [102]. However, the number of reliable markers established to date for the guar genome is still much less in comparison to other legume crops with sequenced genomes. Obviously, this selection of available markers is still insufficient for next-generation breeding programs [99,103,104]. On the other hand, the clear genotypic differences in the stress tolerance patterns of individual guar cultivars indicate their value as a source of valuable genes for breeding for this trait. Therefore, both genomics and transcriptomics data acquired for the drought-tolerant guar plants might deliver important markers for breeding new cultivars with improved drought tolerance [15,105].

For example, a meta-analysis of microsatellite SSR markers accomplished by Pareek and co-workers delivered several genes promising for prospective guar breeding programs, including work on new drought-tolerant cultivars [98]. The transcriptome database established by Thakur and co-workers by sequencing of the total mRNA pool in two guar varieties, RGC-1066 and M-83, contained 5773 potential microsatellite SSR- and 3594 SNP-based markers associated with the genes involved in stress tolerance [106]. Importantly, despite efforts to establish genomic resources in guar, to date, no Quantitative Trait Loci (QTLs) or Genome-wide Association Study (GWAS) has been reported in guar [18].

#### 2.4.2. The Role of Transcriptomics Data in Breeding of New Drought-Tolerant Cultivars

Transcriptomics represents another highly efficient approach currently employed for the search of prospective biotechnology-relevant markers to establish new cultivars with high gum contents [107,108,109]. Thus, post-genomic studies combining transcriptomics and metabolomics platforms emerged over the last decade. Such works are recognized as an important source of valuable information on biomarkers that can be used as a tool for guar breeding [110,111,112].

Another promising approach to address guar stress tolerance is RNA sequencing in combination with de novo transcriptome analysis [113]. For example, Ansari and co-workers reported a de novo comparative transcriptomics survey accomplished with the drought-tolerant cultivar RGC-1002 and the drought-sensitive one RGC-1066 after their exposure to a 30-day drought [34]. The analysis revealed 5206 and 2793 genes, which were drought-dependently up- and down-regulated, respectively, in the tissues of the drought-sensitive RGC-1066 plants. Specifically, the genes encoding enzymes of the transketolase family, phosphoenolpyruvate carboxylase 3, temperature-inducible lipocalin and cytochrome oxidase showed a stress-induced expression enhancement in the drought-tolerant cultivar. The KEGG (Kyoto Encyclopedia of Genes and Genomes)-based bioinformatics analysis revealed up-regulation of the genes related to the starch and sucrose metabolism as the primary molecular basis of the drought tolerance characteristic for the RGC-1002 genotype [34].

To date, RNA sequencing is believed to be one of the most common approaches to identifying the genes critically involved in stress-related metabolic pathways, regulatory events and biological processes. Thus, Alshamery and co-workers performed RNA-Seq by expectation–maximization (RSEM) experiments with a drought-tolerant cultivar BWP-5595 upon a 35-day long drought applied in parallel to regularly watered controls [72]. These kinds of experiments were introduced by Li and Dewey earlier [114]. Further empirical data processing revealed 499 and 191 drought-specifically up- and down-regulated genes, respectively. It was essentially more than reported previously by Jin et al., who identified 32 up- and six down-regulated genes by means of the PlantTFDB database [115]. Among the genes identified in the study of Alshameri and co-workers [72], those encoding the proteins of the proton-dependent oligopeptide transporter and transferase families, aquaporins, calcium/calmodulin-dependent/calcium-dependent protein kinases, asparagine A1 peptidases, UDP-glucuronosyl/UDP-glucosyltransferases and major intrinsic protein (MIP) demonstrated the most pronounced drought-induced increase in the expression levels.

The described response of the guar transcriptome to drought can be considered typical for legumes. Indeed, several well-characterized members of the proton-dependent oligopeptide transporter family are known to be nitrate transporters and play an important role in drought tolerance [116,117]. Aquaporins and MIP proteins are the transmembrane channels and also impact the plant’s tolerance to abiotic stresses, including drought [118]. Asparagine peptidases are well-known plant proteolytic enzymes that are involved in drought protection via abscisic acid (ABA) signaling in guard cells [119]. Over-expression of the aspartic protease gene *ASP G 1* (aspartic protease guard cell 1) in the stomatal cells resulted in enhanced drought tolerance in Arabidopsis plants [119]. *UDP-glucuronosyl/UDP-glucosyltransferases* represent a superfamily of enzymes catalyzing the binding of a glycosyl group to a UTP-sugar molecule [120]. These transferases control the glycosylation of anthocyanine aglycons, modulating, thereby, the accumulation of soluble polyphenols in plant tissues [120,121].

Alshameri and colleagues analyzed the pattern of increased expression of drought-induced transcriptome changes and revealed a prospective increase in abundance and/or activity of 88 enzymes representing 77 KEGG pathways [72]. This was accompanied by up-regulation of MYB- and ERF-related families of transcription factors, which essentially impact the regulation of plant growth, development and responses to abiotic stress. Analysis of drought-induced differential gene expression patterns in the drought-resistant guar cultivar BWP-5595 revealed a total of 12 polypeptides representing six families of transcription factors, which were characterized by a significant decrease in expression levels [72]. Four of them were associated with *bHLH* (basic helix–loop–helix) transcription factors, which are commonly involved in developmental processes (including cell proliferation and differentiation), trichome formation and tolerance to biotic and abiotic stresses [122].

RNA sequencing was also used to investigate the molecular mechanisms underlying drought tolerance in guar, with increased expression levels of genes controlling wax biosynthesis, encoding ATP-binding cassette (*ABC*) transporters; transcription factors of the *NAC*, *WRKY*, *GRAS*, *MYB* families; and a number of previously unknown transcription factor genes in leaves of drought-tolerant guar plants of the RGC-1025 cultivar [123]. The authors were able to demonstrate that enhanced wax deposition on the leaf surface plays a crucial role in enhancing the drought tolerance of guar plants, and knowledge of differentially expressed gene (DEG) patterns allowed the mapping of key genes involved in the wax biosynthesis pathway [123]. In addition, drought-induced up-regulation of genes prospectively involved in response to environmental stress was observed, particularly genes encoding DNA helicases, malate dehydrogenase (*MDH*), aldo-ketoreductase (AKR1), late embryogenesis abundant protein 14 (*LEA14*), pyruvate dehydrogenase (*PDH*) and serine hydroxyl methyltransferase (*SHMT*), as well as increased activity of enzymes involved in detoxification of ROS [123].

Recently, using RNA sequencing coupled with transcriptome analysis, Jaiswal et al. identified four genes encoding metallothioneins (*CtMTs*), which are known to affect ROS protection and heavy metal buffering/detoxification as well as drought stress tolerance [124]. The sequences of three *CtMTs* of guar were similar to metallothioneins of other plants and showed tissue-specific expression in leaves and seeds, whereas the sequence of *CtMT1* contained a unique C-X-C motif. High expression of the *CtMT1* gene was observed in roots and nodules, whereas *CtMT2* and *CtMT3* genes showed high expression in leaves. The expression of the *CtMT4* gene was high in seeds. The qRT-PCR studies revealed upregulation in the expression of the *CtMT1* gene under drought stress.

Recently, Chaudhary et al. reported the identification of guar microRNAs using a bean EST (Expressed Sequence Tags) cluster database and an in silico approach [125]. This approach seems to be very promising for studying drought tolerance in guar, as it gives access to the prediction of the targets related to individual metabolic pathways and regulatory systems, especially those involved in the response to various biotic and environmental stresses [125].

Thus, the problem of guar tolerance to different environmental stresses in general and drought in particular can be addressed only by consideration of integrated genomics, metabolomics, transcriptomics and, ideally, proteomics datasets. Identification of the corresponding biomarkers can be the first step in breeding this important legume crop.

### 2.5. Effect of Drought on the Legume-Microbial Symbiosis in Guar

The tolerance of legume plants to nutritional stresses cannot be addressed without consideration of the rhizosphere microbiome, the most important of which are root microsymbionts, dominating with nodule rhizobia bacteria [126]. It is well known that dehydration is accompanied by pronounced metabolic changes in legume nodules [127]. Nitrogen-fixation capacity of guar is estimated to reach 34 to 54 kg of nitrogen per ha of soil per year [128]. It is important to take into account, however, that the introduction of the crop in new areas requires inoculants, which are typically not available in the soils where the species was not cultured before [129].

Probing the strains available in the U.S. Department of Agriculture’s (USDA-ARS) national *Rhizobium* germplasm collection [130] revealed four *Rhizobium* strains interacting with guar. Inoculation with preparations containing these strains revealed positive effects on multiple traits. Thus, the increase in total assimilated nitrogen, biomass and protein yields was increased in inoculated plants [131]. For example, inoculation with active *Rhizobium* strains under conditions of Sicilia resulted in a 34% increase in seed yield, although the yields of galactomannans were not affected [132]. For Indian soils, inoculation of guar with active strains of *Bradyryrhizobium* spp. was reported. Under those conditions, inoculation resulted in a 28% increase in seed protein yields [133]. When guar was introduced in Sudan, inoculation with *Bradyryrhizobium* spp. strains had a positive effect on plant development, significantly increasing nodule number, plant dry weight, total nitrogen contents and seed yields in comparison to uninoculated plants [134]. It can be assumed that, similarly to other legumes, guar plants have differential responsivity to individual rhizobia strains, i.e., inoculation with different rhizobia might result in different seed productivity gains [135]. However, this aspect remains unaddressed so far.

The ability of guar to form a symbiosis with the fast-growing and relatively unspecific rhizobia *Ensifer aridi* (family *Rhizobiaceae*) was described recently. This rhizobia species has a wide range of legume host plants from the subfamilies *Mimosoideae* and *Papilionoideae* [136].

Recently, guar plants grown in different soils in Russia were inoculated with the strains of nodule bacteria *Bradyrhizobium retamae* RCAM05275 and *Ensifer aridi* RCAM05276 [129]. These strains were isolated from the root nodules of the guar plants grown in Indian soils. These were patented by the authors and deposited in the Departmental Collection of Beneficial Agricultural Microorganisms in the All-Russia Research Institute of Agricultural Microbiology (ARRIAM, St. Petersburg, Russia). Both strains yielded efficient symbiosis with guar in laboratory experiments. Therefore, they are considered for further evaluation and addressed in comprehensive field experiments to establish biopreparations promising in improving the nitrogen supply of guar plants. These experiments revealed an increase in total nitrogen contents (compared to the non-inoculated controls) by about 1.4-fold and nitrogen accumulation in shoots by 3–4-fold [129,137]. These facts indicate the high efficiency of symbiosis with representatives of these bacterial species [129,137].

The effect of water stress on guar root nodules was only minimally addressed in literature so far. Importantly, guar is featured with significantly higher efficiency of nodule formation (nodulation efficiency) under dry and hot conditions in comparison to other legumes [9]. The data regarding the effect of drought on the efficiency of N_2_ fixation in guar nodules sometimes appear contradictory. Venkateswarlu and co-workers reported that water stress applied for eight days (watering interruption) on the 30th 30 days after sowing (vegetative stage) and 50 days from sowing (the flowering) caused a 64.5% and 54.4% reduction in guar nodule fresh weights, respectively, although the stress had no impact on nodule numbers [138]. The authors also found a sharp stress-induced decline in nitrogenase activity when the loss of relative water contents in nodules exceeded 10% [138]. It was also shown that even a slight reduction in water supply could result in a significant decrease in the nodule nitrogenase activity. A seven-day-long drought applied to field-grown guar plants on the 30th day after germination (DAG) caused a 45% drop in the number of nodules and an 80% decrease in their fresh weights in comparison to the irrigated controls [139].

Recently, in greenhouse studies with guar grown in two soil types, Shresta and co-workers showed that different reductions in water supply during 50 days had the greatest negative impact on nodule weights, followed by shoot biomass, number of reproductive nodes, plant height and nodule number [140]. The response (i.e., alteration in nodule weights and shoot biomass values) differed between soils. However, after re-watering, both nodule development and biomass production strongly and rapidly recovered. Therefore, it was concluded that the relatively minor effect on nodule number indicated that the basic machinery for biological nitrogen fixation (nodules) remained largely intact until water stress became extreme. These results show that nodule growth in guar is highly sensitive to water stress, but the plant is resilient in maintaining nodules under water stress and recovering nodule growth upon moisture restoration [140]. However, severe water stress can cause irreversible disruption of biological nitrogen fixation [141], although some legumes, including horse bean, vigna, groundnut, guar and soybean, show more pronounced drought tolerance and generally higher potential for post-stress recovery [138,141,142].

In general, most of the reported studies addressed the ability of guar to retain nodules under moderate (not severe) water stress conditions and to restore their growth and functional activity after resumption of irrigation. This shows that the efficiency of nitrogen-fixing symbiosis under stressed conditions depends on the plant genotype, inoculant strain, duration of stressor exposure and a number of other factors. These factors differently affect the parameters of plant growth, productivity and the state of nitrogen-fixing apparatus [140].

Besides nodule rhizobia, guar can form a symbiotic association with arbuscular mycorrhiza (AM) fungi. AM fungi are the microsymbionts, which are characteristic of 80% of terrestrial plants, including legumes. Specifically, guar forms symbiosis with the fungi of the *Glomus* genus. To date, the biology of this symbiosis remains mostly unstudied. It is well known that mycorrhization of legume crops leads to a pronounced increase in seed yields, protein, carbohydrate, fat and starch contents. It is highly likely that symbiosis with AM fungi might result in enhanced gum production and its higher contents in seeds, although this aspect has not been experimentally addressed so far. For example, inoculation of pea genotypes with maximal symbiosis efficiency with multicomponent formulations containing rhizobia and AM had an effect equivalent to a full dose of mineral fertilizer application [143,144]. Additionally, AM was repeatedly shown to increase plant tolerance to drought via modulation of the activities of antioxidant enzymes, photosynthesis and osmotic adaptation [145,146].

The interaction of guar with both native AM and AM-based preparations has not been studied yet. We could find the only report on guar inoculation with AM-based microbial preparations [8]. The authors described an increase in plant height, root length, number of branches, plant dry weight, leaf area index, chlorophyll contents and plant nutrient uptake compared to the uninoculated control. Seed productivity and quality with respect to seed contents of major metabolites were also increased. In addition, activation of the soil microorganisms in the guar rhizosphere was observed, i.e., the activities of the key enzymes, dehydrogenases, phosphatases, proteases and invertases, were increased in the rhizosphere due to increased bacterial counts and the enhancement of enzyme biosynthesis. However, the fact that mycorrhizal inoculation affects the stress tolerance of guar was not confirmed in that work [8]. Thus, although the necessity to increase the efficiency of guar symbioses with rhizobia and AM fungi is well recognized, the development of highly efficient inoculants for cultivars grown in semi-arid regions remains an urgent need [138].

Table 3 shows a brief summary of drought effects on the key characteristics of guar discussed in this review.

## 3. Conclusions

A comprehensive literature mining clearly indicates that the world genetic resources of *Cyamopsis tetragonoloba* (L.) Taub. are not yet sufficiently characterized with respect to drought tolerance, although an impressive body of data on genotypic variability in this trait, along with underlying mechanisms, is available. Therefore, it appears to be possible to identify the material for breeding drought-tolerant varieties, which is available in gene banks worldwide. On the other hand, it should be recognized that the metabolic features underlying these differences at the metabolome and proteome levels remain poorly understood and mostly unknown. Experiments on modeling water stress (e.g., via interruption of irrigation) under different soil and climate conditions convincingly demonstrated the negative impact of drought on different productivity traits of guar. Therefore, the studies of guar responses to drought might allow for the development of efficient culturing and irrigation protocols for adaptation and introduction in guar practice. This is especially relevant for Russia, where guar was only recently introduced as an object of crop production.

The obtained knowledge might also allow for the adjustment of the irrigation setup (e.g., switch to limited water supply or temporary arrest of irrigation) for the most efficient and economically reasonable guar cultivation. In this context, the search for drought-tolerant genotypes is particularly relevant. The guar genomic resources, which are still very limited, should serve as a compulsory platform for accelerated breeding. Studies of the guar transcriptome, including drought-tolerant genotypes, delivered rich information on prospective transcript-based breeding markers. Differentially expressed genes have been identified, the analysis of which will help to decipher the metabolic pathways involved in plant resistance to drought, find drought-susceptible transcription factors and identify DNA-based markers. This would open broad opportunities for molecular breeding for stress tolerance. Complementation of the DNA- and RNA-based markers with those delivered by proteomics and metabolomics would increase the potential of this strategy.

## Figures and Tables

**Table 1:** Guar accessions from different gene banks worldwide identified as drought tolerant.

S.No.	Accession Name	Country of GenBank	Reference
1	Lewis	The USA *	[15]
2	Santa-Cruz	The USA	[15]
3	Esser	The USA	[31]
4	S-807	The USA	[31]
5	Hall	The USA	[26]
6	PI 323083	The USA	[32]
7	HG-75	India **	[26]
8	RGC-986	India	[33]
9	HG-563	India	[33]
10	RGC-1002	India	[34]
11	RGC-1025.	India	[27]
12	BWP 5595	Pakistan ***	[13,27]
13	BWP 5596	Pakistan	[13]
14	BWP 5597	Pakistan	[13]
15	BWP 5599	Pakistan	[13,27]
16	BWP 5609,	Pakistan	[13]
17	BWP 5611	Pakistan	[13]
18	BR 99	Pakistan	[13]
19	Chiniot 1	Pakistan	[13,27]
20	Chiniot 2	Pakistan	[27]
21	Chiniot Black	Pakistan	[13]
22	Kaloorkot 2	Pakistan	[27]
23	24320	Pakistan	[27]
24	24323	Pakistan	[13]
25	Khushab Black	Pakistan	[13]
26	S-5744	Pakistan	[14]
27	S-5824	Pakistan	[14]

* Plant Genetic Resources Conservation Unit: Griffin, GA. ** National Bureau of Plant Genetic Resources (NBPGR, New Delhi). *** National Agricultural Research Centre (NARC, Islamabad).

**Table 2:** Guar accessions from different gene banks worldwide identified as drought-sensitive.

S.No.	Accession Name	Country of GenBank	Reference
1	S-1183	USA	[31]
2	Brooks	USA	[31]
3	RGC-471	India	[33]
4	Varsha	India	[33]
5	RGC-936	India	[35]
6	RGC-1066	India	[34]
7	24321	Pakistan	[13,27]
8	24332	Pakistan	[27]
9	24333	Pakistan	[27]
10	027340	Pakistan	[27]
11	Bhawana 2	Pakistan	[27]
12	Br 90	Pakistan	[27]
13	Khanewal Local 2	Pakistan	[13,27]
14	Khushab White,	Pakistan	[13]
15	Silanwali White,	Pakistan	[13]
16	Sialkot Black	Pakistan	[13]
17	S-1183	USA	[31]
18	Brooks	USA	[31]

**Table 3:** Summary of drought stress effects on the important characteristics of guar.

S.No	The Trait	Change	Reference
Morphological and agronomic traits			
1	The number of pods per plant	decreased	[15,26,27]
2	Seed mass per plant	decreased	[15,147]
3	Plant height	decreased	[15,27,140]
4	Seeds per pod	was not affected	[15,24]
5	Seed yields	decreased	[15,26,147]
6	Numbers of leaves	decreased	[27]
7	Leaf area	decreased	[27]
8	Leaf area index	decreased	[15]
9	Leaf dry weight	decreased	[27]
10	Total shoot biomass	decreased	[27,140]
11	Gum yields	decreased	[147]
12	Number of reproductive nodes	decreased	[140]
Physiological and biochemical traits			
13	CO_2_ assimilation rate, transpiration rate and air CO_2_ concentration	decreased	[13]
14	Photosynthetic Rates	decreased	[33,35]
15	Nitrogen concentration	decreased significantly	[78]
16	Carbon concentration	increased significantly	[78]
17	NDVI index	increased	[57]
18	Water use efficiency	increased	[15]
19	The contents of carotenoids and anthocyanins	increased	[57]
20	The contents of nicotinamide adenine dinucleotide phosphate (NADPH)	decreased	[33]
21	Biosynthesis of glucose, fructose and fructones	increased	[33]
22	Aspartate aminotransferase (AspAT) and isocitrate dehydrogenase (ICDH) activities	decreased	[78]
23	Aminase and glutamate deaminase (NADH-GDH/NAD-GDH) activities	increased	[78]
24	Levels of the oxidative stress marker malondialdehyde and the total phenolic contents	increased	[35]
25	Activity of enzymes involved in detoxification of ROS	increased	[123]
Molecular and genetic traits			
26	Level of transcripts related to starch and sucrose metabolism	increased	[34]
27	Expression levels of three genes of the *SWEET* family of sugar transporters	increased	[27,72]
28	Transcript levels of the genes related to nitrogen metabolism	decreased	[78,83]
29	Transcript levels of the gene encoding phosphoenolpyruvate carboxylase (*PEPC*)	increased	[78,83]
30	expression level of dihydrolipoamide dehydrogenase (*DLD*)	increased	[78]
31	Transcript level of the key tricarboxylic acid (TCA) components	decreased	[78,83]
32	Expression levels of fumarate dehydrogenase (*FDH*) gene	expressed differentially	[78,83]
33	The level of expression of genes encoding the proteins of the proton-dependent oligopeptide transporter and transferase families, aquaporins, calcium/calmodulin-dependent/calcium-dependent protein kinases, asparagine A1 peptidases, UDP-glucuronosyl/UDP-glucosyltransferases and major intrinsic protein (*MIP*)	increased	[72]
34	Abundance and/or activity of 88 enzymes representing 77 KEGG pathways	increased	[72]
35	Expression levels of genes controlling wax biosynthesis; encoding ATP-binding cassette (*ABC*) transporters; transcription factors of the *NAC*, *WRKY*, *GRAS*, *MYB* families	increased	[123]
36	Expression of *CtMT1* gene	increased	[124]
Symbiotic traits			
37	Nodule fresh weights	decreased	[138,139,140]
38	Nodule numbers	was not affected	[138]
39	Nodule numbers	decreased	[139,140]
40	Nitrogenase activity	decreased	[138]

## References

[1] Chudzikowski R.J. (1971). Guar gum and its applications. J. Soc. Cosmet Chem..

[2] Thombare N., Jha U., Mishra S., Siddiqui M.Z. (2016). Guar gum as a promising starting material for diverse applications: A review. Int. J. Biol. Macromol..

[3] Mudgil D., Barak S., Khatkar B.S. (2014). Guar gum: Processing, properties and food applications-A Review. J. Food Sci. Technol..

[4] Europe Guar Market Size & Share Analysis—Industry Research Report—Growth Trends. https://www.mordorintelligence.com/industry-reports/europe-guar-market.

[5] Dzyubenko E.A., Safronova V.I., Vishnyakova M.A. (2023). Objectives of guar breeding in the Russian Federation in connection with the prospects of domestic guar gum production (review). Sel’skokhozyaistvennaya Biol. [Agric. Biol.].

[6] Garkusha S., Tesheva S., Pischenko D. Evaluation of productivity and quality of rice varieties included into State register of breeding achievements approved for use, in production tests in Krasnodar region. Proceedings of the International Conference on Advances in Agrobusiness and Biotechnology Research (ABR 2021).

[7] Dzyubenko N.I., Dzyubenko E.A., Potokina E., Bulyntsev S.V. (2017). Clusterbeans *Cyamopsis tetragonoloba* (L.) taub.—Properties, use, plant genetic resources and expected introduction in Russia (review). Sel’skokhozyaistvennaya Biol..

[8] El-Sawah A., El-Keblawy A., Ali D., Ibrahim H., El-Sheikh M., Sharma A., Hamoud Y., Shaghaleh H., Brestic M., Skalicky M. (2021). Arbuscular Mycorrhizal Fungi and Plant Growth-Promoting Rhizobacteria Enhance Soil Key Enzymes, Plant Growth, Seed Yield, and Qualitative Attributes of Guar. Agriculture.

[9] Zahran H.H. (1999). Rhizobium-legume symbiosis and nitrogen fixation under severe conditions and in an arid climate. Microbiol. Mol. Biol. Rev..

[10] Khanna-Chopra R., Singh K., Tripathi B.N., Müller M. (2015). Drought Resistance in Crops: Physiological and Genetic Basis of Traits for Crop Productivity. Stress Responses in Plants: Mechanisms of Toxicity and Tolerance.

[11] Undersander D.J., Putnam D.H., Kaminski A.R., Kelling K.A., Doll J.D., Oplinger E.S., Gunsolus J.L. Alternative Field Crops Manual; Guar. https://www.hort.purdue.edu/newcrop/afcm/guar.html.

[12] Sultan M., Zakir N., Rabbani M.A., Shinwari Z.K., Masood M.S. (2013). Genetic diversity of guar (*Cyamopsis tetragonoloba* L.) landraces from Pakistan based on RAPD markers. Pak. J. Bot..

[13] Ali Z., Ashraf M., Al-Qurainy F., Khan S., Akram N.A. (2015). Field screening of guar (*Cyamopsis tetragonoloba* L.). Pak. J. Bot..

[14] Zubair M., Akhtar L.H., Minhas R., Qamar M.J., Bukhari S.J., Sadiq A., Hussain S., Ammar A., Aziz M.K. (2017). Expediency of water and soil nutrients in irrigated and extreme drought conditions. AJAR.

[15] Avola G., Riggi E., Trostle C., Sortino O., Gresta F. (2020). Deficit Irrigation on Guar Genotypes (*Cyamopsis tetragonoloba* (L.) Taub.): Effects on Seed Yield and Water Use Efficiency. Agronomy.

[16] Lebed D.V., Kostenkova E.V., Voloshin M.I. (2017). Agronomic rationale for the growing of *Cyamopsis tetragonoloba* (L.) in southern European Russia. Tavricheskiy Vestn. Agrar. Nauk. Taurida Her. Agrar. Sci..

[17] Startsev V.I., Livanskaya G.A., Kulikov M.A., Startsev V.I., Livanskaya G.A., Kulikov M.A. (2017). Prospects of cultivation of guar (*Cyamopsis tetragonoloba* L.) in Russia. VRGAZU Sci. J..

[18] Ravelombola W., Manley A., Adams C., Trostle C., Ale S., Shi A., Cason J. (2021). Genetic and genomic resources in guar: A review. Euphytica.

[19] Stafford R.E., Lewis C.R. (1975). Natural Crossing in Guar, *Cyamopsis tetragonoloba* (L.) Taub.1. Crop Sci..

[20] Mangelsdorf P.C. (1952). The Origin, Variation, Immunity and Breeding of Cultivated Plants. N. I. Vavilov; trans. from the Russian by K. Starr Chester. Waltham, Mass.: Chronica Botanica; New York: Stechert-Hafner, 1951. 364 pp. $7.50. Science.

[21] Kumar S., Palve A.S., Patel S.K., Selvanayagam S., Sharma R., Rathore A. (2020). Development of genomic microsatellite markers in cluster bean using next-generation DNA sequencing and their utility in diversity analysis. Curr. Plant Biol..

[22] Srivastava M., Kapoor V.P. (2005). Seed galactomannans: An overview. Chem. Biodivers.

[23] Murwan K.A., Abdalla A.H., Sulafa N. (2012). Compositional Characterization of Endosperm (Guar Gum) of Six Guar (*Cyamopsis tetragonoloba*) Genotypes Grown in Sudan. ISCA J. Biological Sci..

[24] Gresta F., Mercati F., Santonoceto C., Abenavoli M.R., Ceravolo G., Araniti F., Anastasi U., Sunseri F. (2016). Morpho-agronomic and AFLP characterization to explore guar (*Cyamopsis tetragonoloba* L.) genotypes for the Mediterranean environment. Ind. Crops Prod..

[25] Losavio N., Ventrella D., Vonella A.V. (2002). Adattabilità ambientale e potenzialità produttiva del guar coltivato in regime irriguo nell’Italia meridionale. Riv. Agron..

[26] Stafford R.E., McMichael B.L. (1991). Effect of Water Stress on Yield Components in Guar. J. Agron. Crop Sci..

[27] Al-Shameri A., Al-Qurainy F., Khan S., Mohammad N., Gaafar A.-R., Tarroum M., Alameri A., Alansi S., Ashraf M. (2017). Appraisal of guar [*Cyamopsis tetragonoloba* (L.) Taub.] accessions for forage purpose under the typical saudi arabian environmental conditions encompassing high temperature, salinity and drought. Pak. J. Bot..

[28] Ahmed M.F.E.M., Mac D.M., Bashir A.A.G. (2011). Effect of water stress at different periods on seed yield and water use efficiency of guar under Shambat conditions. Agric. Sci..

[29] Meftahizadeh H., Ghorbanpour M., Asareh M.H. (2019). Changes in phenological attributes, yield and phytochemical compositions of guar (*Cyamopsis tetragonoloba* L.) landaraces under various irrigation regimes and planting dates. Sci. Hortic..

[30] Baath G.S., Rocateli A.C., Kakani V.G., Singh H., Northup B.K., Gowda P.H., Katta J.R. (2020). Growth and physiological responses of three warm-season legumes to water stress. Sci. Rep..

[31] Khandaza, Ashraf B., Ala S., Alam S.M., Shirazi M., Ansari R. (2001). Water relations in different gur (*Cyamopsis tetragonoloba* L. Tuab) genotypes under water stress. Pak. J. Bot..

[32] Nanduri K., Shahid M. (2011). Potential of cowpea [*Vigna unguiculata* (L.) Walp.] and guar [*Cyamopsis tetragonoloba* (L.) Taub.] as alternative forage legumes for the United Arab Emirates. Emir. J. Food Agric..

[33] Pandey K., Kumar R.S., Prasad P., Pande V., Trivedi P.K., Shirke P.A. (2022). Coordinated regulation of photosynthesis and sugar metabolism in guar increases tolerance to drought. Environ. Exp. Bot..

[34] Ansari M.A., Bano N., Kumar A., Dubey A.K., Asif M.H., Sanyal I., Pande V., Pandey V. (2022). Comparative transcriptomic analysis and antioxidant defense mechanisms in clusterbean (*Cyamopsis tetragonoloba* (L.) Taub.) genotypes with contrasting drought tolerance. Funct Integr. Genom..

[35] Upreti P., Narayan S., Khan F., Tewari L.M., Shirke P.A. (2021). Drought-induced responses on physiological performance in cluster bean [*Cyamopsis tetragonoloba* (L.) Taub.]. Plant Physiol. Rep..

[36] Osmolovskaya N., Shumilina J., Kim A., Didio A., Grishina T., Bilova T., Keltsieva O.A., Zhukov V., Tikhonovich I., Tarakhovskaya E. (2018). Methodology of Drought Stress Research: Experimental Setup and Physiological Characterization. Int. J. Mol. Sci..

[37] Zhang Q. (2007). Strategies for developing Green Super Rice. Proc. Natl. Acad. Sci. USA.

[38] Fang Y., Xiong L. (2015). General mechanisms of drought response and their application in drought resistance improvement in plants. Cell Mol. Life Sci..

[39] Frolov A., Bilova T., Paudel G., Berger R., Balcke G.U., Birkemeyer C., Wessjohann L.A. (2017). Early responses of mature *Arabidopsis thaliana* plants to reduced water potential in the agar-based polyethylene glycol infusion drought model. J. Plant Physiol..

[40] Kooyers N.J. (2015). The evolution of drought escape and avoidance in natural herbaceous populations. Plant Sci..

[41] Paudel G., Bilova T., Schmidt R., Greifenhagen U., Berger R., Tarakhovskaya E., Stöckhardt S., Balcke G.U., Humbeck K., Brandt W. (2016). Osmotic stress is accompanied by protein glycation in *Arabidopsis thaliana*. J. Exp. Bot..

[42] Ramachandra Reddy A., Chaitanya K.V., Vivekanandan M. (2004). Drought-induced responses of photosynthesis and antioxidant metabolism in higher plants. J. Plant Physiol..

[43] Shi Y., Liu X., Zhao S., Guo Y. (2022). The PYR-PP2C-CKL2 module regulates ABA-mediated actin reorganization during stomatal closure. New Phytol..

[44] Hsu P.-K., Dubeaux G., Takahashi Y., Schroeder J.I. (2021). Signaling mechanisms in abscisic acid-mediated stomatal closure. Plant J..

[45] Liu H., Song S., Zhang H., Li Y., Niu L., Zhang J., Wang W. (2022). Signaling Transduction of ABA, ROS, and Ca^2+^ in Plant Stomatal Closure in Response to Drought. Int. J. Mol. Sci..

[46] Muller B., Pantin F., Génard M., Turc O., Freixes S., Piques M., Gibon Y. (2011). Water deficits uncouple growth from photosynthesis, increase C content, and modify the relationships between C and growth in sink organs. J. Exp. Bot..

[47] González E.M. (2023). Drought Stress Tolerance in Plants. Int. J. Mol. Sci..

[48] Zhang X., Xie Z., Lang D., Chu Y., Cui G., Jia X. (2021). *Bacillus pumilus* improved drought tolerance in *Glycyrrhiza uralensis* G5 seedlings through enhancing primary and secondary metabolisms. Physiol. Plant.

[49] Rodrigues A.M., Jorge T., Osorio S., Pott D.M., Lidon F.C., DaMatta F.M., Marques I., Ribeiro-Barros A.I., Ramalho J.C., António C. (2021). Primary Metabolite Profile Changes in Coffea spp. Promoted by Single and Combined Exposure to Drought and Elevated CO_2_ Concentration. Metabolites.

[50] Doddaraju P., Dharmappa P.M., Thiagarayaselvam A., Vijayaraghavareddy P., Bheemanahalli R., Basavaraddi P.A., Malagondanahalli M.K.V., Kambalimath S., Thulasiram H.V., Sreeman S.M. (2023). Comprehensive analysis of physiological and metabolomic responses to drought reveals specific modulation of acquired tolerance mechanisms in rice. Physiol. Plant.

[51] Wang X., Cai X., Xu C., Wang Q., Dai S. (2016). Drought-Responsive Mechanisms in Plant Leaves Revealed by Proteomics. Int. J. Mol. Sci..

[52] Razi K., Muneer S. (2021). Drought stress-induced physiological mechanisms, signaling pathways and molecular response of chloroplasts in common vegetable crops. Crit. Rev. Biotechnol..

[53] Chaves M.M., Flexas J., Pinheiro C. (2009). Photosynthesis under drought and salt stress: Regulation mechanisms from whole plant to cell. Ann. Bot..

[54] Challabathula D., Zhang Q., Bartels D. (2018). Protection of photosynthesis in desiccation-tolerant resurrection plants. J. Plant Physiol..

[55] Flexas J., Medrano H. (2002). Drought-inhibition of photosynthesis in C3 plants: Stomatal and non-stomatal limitations revisited. Ann. Bot..

[56] Dahal K., Wang J., Martyn G.D., Rahimy F., Vanlerberghe G.C. (2014). Mitochondrial alternative oxidase maintains respiration and preserves photosynthetic capacity during moderate drought in *Nicotiana tabacum*. Plant Physiol..

[57] Upreti P., Narayan S., Khan F., Tewari L.M., Shirke P.A. (2021). Physiological attributes associated with leaf spectral alterations in guar [*Cyamopsis tetragonoloba* (L.) Taub.] under drought. 3 Biotech.

[58] Wong C.Y.S., Gamon J.A. (2015). Three causes of variation in the photochemical reflectance index (PRI) in evergreen conifers. New Phytol..

[59] Singh R., Naskar J., Pathre U.V., Shirke P.A. (2014). Reflectance and cyclic electron flow as an indicator of drought stress in cotton (*Gossypium hirsutum*). Photochem. Photobiol..

[60] Ranjan S., Singh R., Singh M., Pathre U.V., Shirke P.A. (2014). Characterizing photoinhibition and photosynthesis in juvenile-red versus mature-green leaves of *Jatropha curcas* L. Plant Physiol. Biochem..

[61] Verbeke S., Padilla-Díaz C.M., Haesaert G., Steppe K. (2022). Osmotic Adjustment in Wheat (*Triticum aestivum* L.) during Pre- and Post-anthesis Drought. Front. Plant Sci..

[62] Qazi H.A., Srinivasa Rao P., Kashikar A., Suprasanna P., Bhargava S. (2014). Alterations in stem sugar content and metabolism in sorghum genotypes subjected to drought stress. Funct Plant Biol..

[63] Benkeblia N. (2022). Insights on Fructans and Resistance of Plants to Drought Stress. Front. Sustain. Food Syst..

[64] Avigad G., Dey P.M., Dey P.M., Harborne J.B. (1997). 4—Carbohydrate Metabolism: Storage Carbohydrates. Plant Biochemistry.

[65] Tapernoux-Lüthi E.M., Böhm A., Keller F. (2004). Cloning, Functional Expression, and Characterization of the Raffinose Oligosaccharide Chain Elongation Enzyme, Galactan:Galactan Galactosyltransferase, from Common Bugle Leaves. Plant Physiol..

[66] Du Y., Zhao Q., Chen L., Yao X., Zhang W., Zhang B., Xie F. (2020). Effect of drought stress on sugar metabolism in leaves and roots of soybean seedlings. Plant Physiol. Biochem. PPB.

[67] Mathan J., Singh A., Ranjan A. (2021). Sucrose transport in response to drought and salt stress involves ABA-mediated induction of OsSWEET13 and OsSWEET15 in rice. Physiol. Plant.

[68] Cairns A.J., Ashton J.E. (1993). Species-dependent patterns of fructan synthesis by enzymes from excised leaves of oat, wheat, barley and timothy. New Phytol..

[69] Fox H., Ben-Dor S., Doron-Faigenboim A., Goldsmith M., Klein T., David-Schwartz R. (2023). Carbohydrate dynamics in Populus trees under drought: An expression atlas of genes related to sensing, translocation, and metabolism across organs. Physiol. Plant.

[70] Chen T., Zhang Z., Li B., Qin G., Tian S. (2021). Molecular basis for optimizing sugar metabolism and transport during fruit development. aBIOTECH.

[71] Cho Y.-G., Kang K.-K. (2020). Functional Analysis of Starch Metabolism in Plants. Plants.

[72] Alshameri A., Al-Qurainy F., Gaafar A.-R., Khan S., Nadeem M., Alansi S., Shaikhaldein H.O., Salih A.M. (2020). Identification of Differentially Expressed Drought-Responsive Genes in Guar [*Cyamopsis tetragonoloba* (L.) Taub]. Int. J. Genom..

[73] Xu Z., Zhou G. (2004). Research advance in nitrogen metabolism of plant and its environmental regulation. Ying Yong Sheng Tai Xue Bao.

[74] Heinemann B., Hildebrandt T.M. (2021). The role of amino acid metabolism in signaling and metabolic adaptation to stress-induced energy deficiency in plants. J. Exp. Bot..

[75] Yang M., Geng M., Shen P., Chen X., Li Y., Wen X. (2019). Effect of post-silking drought stress on the expression profiles of genes involved in carbon and nitrogen metabolism during leaf senescence in maize (*Zea mays* L.). Plant Physiol. Biochem..

[76] Zhao J.-B., Du C.-J., Ma C.-M., Sun J.-C., Han Z.-T., Yan D.-H., Jiang Z.-P., Shi S.-Q. (2020). Response of photosynthesis and carbon/nitrogen metabolism to drought stress in Chinese chestnut “Yanshanzaofeng” seedlings. Ying Yong Sheng Tai Xue Bao.

[77] Cao X., Zhong C., Zhu C., Zhu L., Zhang J., Wu L., Jin Q. (2018). Ammonium uptake and metabolism alleviate PEG-induced water stress in rice seedlings. Plant Physiol. Biochem..

[78] Pandey K., Pande V., Prasad P., Shirke P., Kumar R.S. (2022). Synchronised interaction of carbon and nitrogen provides drought tolerance in *Cyamopsis tetragonoloba*. Environ. Exp. Bot..

[79] Castañeda V., Gil-Quintana E., Echeverria A., González E.M., Shrawat A., Zayed A., Lightfoot D.A. (2018). Legume Nitrogen Utilization Under Drought Stress. Engineering Nitrogen Utilization in Crop Plants.

[80] Zuo Q., Liu J., Shan J., Zhou J., Wang L., Yang G., Leng S., Liu H. (2019). Carbon and Nitrogen Assimilation and Partitioning in Canola (*Brassica Napus* L.) in Saline Environment. Commun. Soil Sci. Plant Anal..

[81] Sakakibara H., Takei K., Hirose N. (2006). Interactions between nitrogen and cytokinin in the regulation of metabolism and development. Trends Plant Sci..

[82] Reguera M., Peleg Z., Abdel-Tawab Y.M., Tumimbang E.B., Delatorre C.A., Blumwald E. (2013). Stress-induced cytokinin synthesis increases drought tolerance through the coordinated regulation of carbon and nitrogen assimilation in rice. Plant Physiol..

[83] Kishorekumar R., Bulle M., Wany A., Gupta K.J. (2020). An Overview of Important Enzymes Involved in Nitrogen Assimilation of Plants. Methods Mol. Biol..

[84] Zia R., Nawaz M.S., Siddique M.J., Hakim S., Imran A. (2021). Plant survival under drought stress: Implications, adaptive responses, and integrated rhizosphere management strategy for stress mitigation. Microbiol. Res..

[85] Wang X., Li Y., Wang X., Li X., Dong S. (2022). Physiology and metabonomics reveal differences in drought resistance among soybean varieties. Bot. Stud..

[86] Sezgin A., Altuntaş C., Demiralay M., Cinemre S., Terzi R. (2019). Exogenous alpha lipoic acid can stimulate photosystem II activity and the gene expressions of carbon fixation and chlorophyll metabolism enzymes in maize seedlings under drought. J. Plant Physiol..

[87] Atkin O.K., Macherel D. (2009). The crucial role of plant mitochondria in orchestrating drought tolerance. Ann. Bot..

[88] Shi Y., Chang Y.-L., Wu H.-T., Shalmani A., Liu W.-T., Li W.-Q., Xu J.-W., Chen K.-M. (2020). OsRbohB-mediated ROS production plays a crucial role in drought stress tolerance of rice. Plant Cell Rep..

[89] Oliver M.J., Cushman J.C., Koster K.L. (2010). Dehydration tolerance in plants. Methods Mol. Biol..

[90] Qi J., Song C.-P., Wang B., Zhou J., Kangasjärvi J., Zhu J.-K., Gong Z. (2018). Reactive oxygen species signaling and stomatal movement in plant responses to drought stress and pathogen attack. J. Integr. Plant Biol..

[91] Mushtaq N.U., Saleem S., Rasool A., Shah W.H., Tahir I., Hakeem K.R., Rehman R.U., Aftab T., Hakeem K.R. (2022). Functional Characterization of the Antioxidant Enzymes in Plants Exposed to Environmental Stresses. Antioxidant Defense in Plants: Molecular Basis of Regulation.

[92] Du C., Chai L., Wang Z., Fan H. (2019). Response of proteome and morphological structure to short-term drought and subsequent recovery in *Cucumis sativus* leaves. Physiol. Plant.

[93] Muhammad Aslam M., Waseem M., Jakada B.H., Okal E.J., Lei Z., Saqib H.S.A., Yuan W., Xu W., Zhang Q. (2022). Mechanisms of Abscisic Acid-Mediated Drought Stress Responses in Plants. Int. J. Mol. Sci..

[94] Yu Y., Wang P., Bai Y., Wang Y., Wan H., Liu C., Ni Z. (2020). The soybean F-box protein GmFBX176 regulates ABA-mediated responses to drought and salt stress. Environ. Exp. Bot..

[95] Tayyab N., Naz R., Yasmin H., Nosheen A., Keyani R., Sajjad M., Hassan M.N., Roberts T.H. (2020). Combined seed and foliar pre-treatments with exogenous methyl jasmonate and salicylic acid mitigate drought-induced stress in maize. PLoS ONE.

[96] Ramanjaneyulu A., Madhavi A., Venkata Ramana M., Neelima T., Kareddy I., Asalla S., Ghanta A., Sameer Kumar C.V. (2018). Agronomic and Genetic Analysis of Performance of Guar Varieties under Rainfed Conditions in a Semi-Arid Climate on Alfisols. Int. J. Curr. Microbiol. Appl. Sci..

[97] Honnaiah P.A., Sridhara S., Gopakkali P., Ramesh N., Mahmoud E.A., Abdelmohsen S.A.M., Alkallas F.H., El-Ansary D.O., Elansary H.O. (2021). Influence of sowing windows and genotypes on growth, radiation interception, conversion efficiency and yield of guar. Saudi J. Biol. Sci..

[98] Pareek S., Jaiswal P.S., Shrivastava D. (2022). Guar genes to genome and meta-analysis of SSR markers in sequencing studies. Genet Resour. Crop Evol..

[99] Tanwar U.K., Pruthi V., Randhawa G.S. (2017). RNA-Seq of Guar (*Cyamopsis tetragonoloba*, L. Taub.) Leaves: De novo Transcriptome Assembly, Functional Annotation and Development of Genomic Resources. Front. Plant Sci..

[100] Tyagi A., Sandhya, Sharma P., Saxena S., Sharma R., Amitha Mithra S.V., Solanke A.U., Singh N.K., Sharma T.R., Gaikwad K. (2019). The genome size of clusterbean (*Cyamopsis tetragonoloba*) is significantly smaller compared to its wild relatives as estimated by flow cytometry. Gene.

[101] Kaila T., Chaduvla P.K., Rawal H.C., Saxena S., Tyagi A., Mithra S.V.A., Solanke A.U., Kalia P., Sharma T.R., Singh N.K. (2017). Chloroplast Genome Sequence of Clusterbean (*Cyamopsis tetragonoloba* L.): Genome Structure and Comparative Analysis. Genes.

[102] Grigoreva E., Ulianich P., Ben C., Gentzbittel L., Potokina E. (2019). First Insights into the Guar (*Cyamopsis tetragonoloba* (L.) Taub.) Genome of the ‘Vavilovskij 130’ Accession, Using Second and Third-Generation Sequencing Technologies. Russ. J. Genet..

[103] Kumar S., Parekh M.J., Patel C.B., Zala H.N., Sharma R., Kulkarni K.S., Fougat R.S., Bhatt R.K., Sakure A.A. (2016). Development and validation of EST-derived SSR markers and diversity analysis in cluster bean (*Cyamopsis tetragonoloba*). J. Plant Biochem. Biotechnol..

[104] Rawal H.C., Kumar S., Kumar S., Mithra S.V.A., Solanke A.U., Nigam D., Saxena S., Tyagi A., Yadav N.R., Kalia P. (2017). High Quality Unigenes and Microsatellite Markers from Tissue Specific Transcriptome and Development of a Database in Clusterbean (*Cyamopsis tetragonoloba*, L. Taub). Genes.

[105] Hatfield J.L., Dold C. (2019). Water-Use Efficiency: Advances and Challenges in a Changing Climate. Front. Plant Sci..

[106] Thakur O., Randhawa G.S. (2018). Identification and characterization of SSR, SNP and InDel molecular markers from RNA-Seq data of guar (*Cyamopsis tetragonoloba*, L. Taub.) roots. BMC Genom..

[107] Sharma S., Tyagi A., Srivastava H., Ramakrishna G., Sharma P., Sevanthi A.M., Solanke A.U., Sharma R., Singh N.K., Sharma T.R. (2021). Exploring the edible gum (galactomannan) biosynthesis and its regulation during pod developmental stages in clusterbean using comparative transcriptomic approach. Sci. Rep..

[108] Naoumkina M., Vaghchhipawala S., Tang Y., Ben Y., Powell R.J., Dixon R.A. (2008). Metabolic and genetic perturbations accompany the modification of galactomannan in seeds of Medicago truncatula expressing mannan synthase from guar (*Cyamopsis tetragonoloba* L.). Plant Biotechnol. J..

[109] Gao X., Hu X., Mo F., Ding Y., Li M., Li R. (2022). Repellency Mechanism of Natural Guar Gum-Based Film Incorporated with Citral against Brown Planthopper, *Nilaparvata lugens* (Stål) (Hemiptera: Delphacidae). Int. J. Mol. Sci..

[110] Rajaprakasam S., Rahman H., Karunagaran S., Bapu K., J.R., Kulandivelu G., Kambale R., Ramanathan V., Muthurajan R. (2021). Comparative transcriptome and metabolome profiling in the maturing seeds of contrasting cluster bean (*Cyamopsis tetragonoloba* L. Taub) cultivars identified key molecular variations leading to increased gum accumulation. Gene.

[111] Arkhimandritova S., Shavarda A., Potokina E. (2020). Key metabolites associated with the onset of flowering of guar genotypes (*Cyamopsis tetragonoloba* (L.) Taub). BMC Plant Biol..

[112] Grigoreva E., Tkachenko A., Arkhimandritova S., Beatovic A., Ulianich P., Volkov V., Karzhaev D., Ben C., Gentzbittel L., Potokina E. (2021). Identification of Key Metabolic Pathways and Biomarkers Underlying Flowering Time of Guar (*Cyamopsis tetragonoloba* (L.) Taub.) via Integrated Transcriptome-Metabolome Analysis. Genes.

[113] Al-Qurainy F., Alshameri A., Gaafar A.-R., Khan S., Nadeem M., Alameri A.A., Tarroum M., Ashraf M. (2019). Comprehensive Stress-Based De Novo Transcriptome Assembly and Annotation of Guar (*Cyamopsis tetragonoloba* (L.) Taub.): An Important Industrial and Forage Crop. Int. J. Genom..

[114] Li B., Dewey C.N. (2011). RSEM: Accurate transcript quantification from RNA-Seq data with or without a reference genome. BMC Bioinform..

[115] Jin J., Tian F., Yang D.-C., Meng Y.-Q., Kong L., Luo J., Gao G. (2017). PlantTFDB 4.0: Toward a central hub for transcription factors and regulatory interactions in plants. Nucleic Acids Res..

[116] Tsay Y.-F., Chiu C.-C., Tsai C.-B., Ho C.-H., Hsu P.-K. (2007). Nitrate transporters and peptide transporters. FEBS Lett..

[117] Gu C., Song A., Zhang X., Wang H., Li T., Chen Y., Jiang J., Chen F., Chen S. (2016). Cloning of chrysanthemum high-affinity nitrate transporter family (CmNRT2) and characterization of CmNRT2.1. Sci. Rep..

[118] Sui H., Han B.G., Lee J.K., Walian P., Jap B.K. (2001). Structural basis of water-specific transport through the AQP1 water channel. Nature.

[119] Yao X., Xiong W., Ye T., Wu Y. (2012). Overexpression of the aspartic protease *ASPG1* gene confers drought avoidance in *Arabidopsis*. J. Exp. Bot..

[120] Li P., Li Y.-J., Zhang F.-J., Zhang G.-Z., Jiang X.-Y., Yu H.-M., Hou B.-K. (2017). The Arabidopsis UDP-glycosyltransferases UGT79B2 and UGT79B3, contribute to cold, salt and drought stress tolerance via modulating anthocyanin accumulation. Plant J..

[121] Li T., Xu X., Li Y., Wang H., Li Z., Li Z. (2015). Comparative transcriptome analysis reveals differential transcription in heat-susceptible and heat-tolerant pepper (*Capsicum annum* L.) cultivars under heat stress. J. Plant Biol..

[122] Liu R., Wang Y., Tang S., Cai J., Liu S., Zheng P., Sun B. (2021). Genome-wide identification of the tea plant bHLH transcription factor family and discovery of candidate regulators of trichome formation. Sci. Rep..

[123] Reddy B.M., Anthony Johnson A.M., Jagadeesh Kumar N., Venkatesh B., Jayamma N., Pandurangaiah M., Sudhakar C. (2022). De novo Transcriptome Analysis of Drought-Adapted Cluster Bean (Cultivar RGC-1025) Reveals the Wax Regulatory Genes Involved in Drought Resistance. Front. Plant Sci..

[124] Jaiswal P.S., Mittal N., Randhawa G.S. (2018). *Cyamopsis tetragonoloba* type 1 metallothionein (*CtMT1*) gene is upregulated under drought stress and its protein product has an additional C-X-C motif and unique metal binding pattern. Int. J. Biol. Macromol..

[125] Chaudhary V., Jangra S., Yadav N.R. (2022). In silico Identification of miRNAs and Their Targets in Cluster Bean for Their Role in Development and Physiological Responses. Front. Genet..

[126] Valentine A.J., Benedito V.A., Kang Y. (2018). Legume Nitrogen Fixation and Soil Abiotic Stress: From Physiology to Genomics and Beyond. Annual Plant Reviews Online.

[127] Soboleva A., Frolova N., Bureiko K., Shumilina J., Balcke G., Zhukov V., Tikhonovich I., Frolov A. (2022). Dynamics of Reactive Carbonyl Species in Pea Root Nodules in Response to Polyethylene Glycol (PEG)-Induced Osmotic Stress. Int. J. Mol. Sci..

[128] Mubarak A.R., Salih N.O.H., Hassabo A. (2015). Fate of 15N-labeled urea under a guar-wheat rotation as influenced by crop residue incorporation in a semi-arid Vertisol. Trop. Agric..

[129] Ulianich P.S. (2022). Effectiveness of nitrogen-fixing symbiosis of guar (*Cyamopsis tetragonoloba*) with strains Bradyrhizobium retamae RCAM05275 and Ensifer aridi RCAM05276 in pot experiment. Sel’skokhozyaistvennaya Biol..

[130] USDA ARS National Rhizobium Germplasm Collection|Ag Data Commons. https://data.nal.usda.gov/dataset/usda-ars-national-rhizobium-germplasm-collection.

[131] MacMillan J., Adams C.B., Trostle C., Rajan N. (2021). Testing the efficacy of existing USDA Rhizobium germplasm collection accessions as inoculants for guar. Ind. Crops Prod..

[132] Gresta F., Trostle C., Sortino O., Santonoceto C., Avola G. (2019). Rhizobium inoculation and phosphate fertilization effects on productive and qualitative traits of guar (*Cyamopsis tetragonoloba* (L.) Taub.). Ind. Crops Prod..

[133] Singh R.V., Singh R.R. (1989). Effect of nitrogen, phosphorus and seeding rates on yield, nutrient uptake and water use of guar under dryland conditions. Ann. Agric. Res..

[134] Elsheikh E.A.E., Ibrahim K.A. (1999). The effect of Bradyrhizobium inoculation on yield and seed quality of guar (*Cyamopsis tetragonoloba* L.). Food Chem..

[135] Mamontova T., Afonin A.M., Ihling C., Soboleva A., Lukasheva E., Sulima A.S., Shtark O.Y., Akhtemova G.A., Povydysh M.N., Sinz A. (2019). Profiling of Seed Proteome in Pea (*Pisum sativum* L.) Lines Characterized with High and Low Responsivity to Combined Inoculation with Nodule Bacteria and Arbuscular Mycorrhizal Fungi. Molecules.

[136] Quéré A.L., Tak N., Gehlot H.S., Lavire C., Meyer T., Chapulliot D., Rathi S., Sakrouhi I., Rocha G., Rohmer M. (2017). Genomic characterization of Ensifer aridi, a proposed new species of nitrogen-fixing rhizobium recovered from Asian, African and American deserts. BMC Genom..

[137] Kuznetsova I.G., Sazanova A., Safronova V., Popova J.P., Sokolova D., Tikhomirova N.Y., Osledkin Y.S., Karlov D.S., Belimov A. (2018). Isolation and identification of root nodule bacteria from guar *Cyamopsis tetragonoloba* (L.) Taub. Sel’skokhozyaistvennaya Biol..

[138] Venkateswarlu B., Rao A.V., Lahiri A.N. (1983). Effect of water stress on nodulation and nitrogenase activity of guar (*Cyamopsis tetragonoloba* (L.) Taub.). Proc. Indian Acad. Sci..

[139] Rao A.V., Venkateswarlu B. (1987). Nitrogen fixation as influenced by water stress in selected crop legumes of the Indian Arid Zone. Arid. Soil Res. Rehabil..

[140] Shrestha R., Adams C.B., Rajan N. (2022). Does the drought tolerance of guar [*Cyamopsis tetragonoloba* (L.) Taub.] extend belowground to root nodules?. J. Agron. Crop Sci..

[141] Hungria M., Vargas M.A.T. (2000). Environmental factors affecting N_2_ fixation in grain legumes in the tropics, with an emphasis on Brazil. Field Crops Res..

[142] Subbarao G.V., Johansen C., Slinkard A.E., Nageswara Rao R.C., Saxena N.P., Chauhan Y.S., Lawn R.J. (1995). Strategies for Improving Drought Resistance in Grain Legumes. Crit. Rev. Plant Sci..

[143] Shtark O.Y., Danilova T.N., Naumkina T.S., Vasilchikov A.G., Chebotar V.K., Kazakov A.E., Zhernakov A.I., Nemankin T.A., Prilepskaya N.A., Borisov A.U. (2006). Analysis Of Pea (*Pisum sativum* L.) Source Material For Breeding of Cultivars with High Symbiotic Potential and Choice of Criteria for Its Evaluation. Ecol. Genet..

[144] Intimate Associations of Beneficial Soil Microbes with Host Plants|SpringerLink. https://link.springer.com/chapter/10.1007/978-90-481-9479-7_5.

[145] Wu Q.-S., Zou Y.-N. (2017). Arbuscular Mycorrhizal Fungi and Tolerance of Drought Stress in Plants. Arbuscular Mycorrhizas and Stress Tolerance of Plants.

[146] Frontiers|Arbuscular Mycorrhizal Fungi Alleviate Drought Stress in C3 (Leymus chinensis) and C4 (Hemarthria altissima) Grasses via Altering Antioxidant Enzyme Activities and Photosynthesis. https://www.frontiersin.org/articles/10.3389/fpls.2019.00499/full.

[147] Kruchina-Bogdanov I., Miroshnichenko E., Shaukharov R., Kantemirova E., Golovina M., Abdullaev K., Balashov A., Rusinova E., Rusinov P., Potokina E. (2019). Impact of growing conditions on the gum properties of different genotypes of guar (*Cyamopsis tetragonoloba* (L.) Taub.). Vavilov J. Genet. Breed..

